# Using Anderson's Model of Health Service Utilization to Assess the Use of HIV Testing Services by Sexually Active Men in Ghana

**DOI:** 10.3389/fpubh.2020.00512

**Published:** 2020-09-15

**Authors:** Abdul-Aziz Seidu

**Affiliations:** ^1^Department of Population and Health, University of Cape Coast, Cape Coast, Ghana; ^2^College of Public Health, Medical and Veterinary Sciences, James Cook University, Townsville, QLD, Australia

**Keywords:** men, Ghana, Anderson model, HIV/AIDS, health service utilization, global health, public health, model

## Abstract

**Introduction:** Globally, HIV testing and counseling is considered a key cost-effective component of HIV prevention and treatment. This study sought to use Anderson's model of health service utilization to assess the uptake of HIV testing services by sexually active men in Ghana.

**Materials and Methods:** Data were from the 2014 Ghana Demographic and Health Survey. Both bivariate and multivariate analysis were conducted. The multivariate analysis results are presented as Adjusted Odds ratios (AORs) with 95% confidence intervals (CI). Statistical significance was declared at *p* < 0.05.

**Results:** A total of 3,052 sexually active men aged 15–59 were included in the analysis. Of these, 25.4% tested for their HIV status. Men aged 30–39 (AOR = 2.715, CI = 1.458, 5.054), those with higher level of education (AOR = 3.566,CI = 2.309, 5.509), married (AOR = 1.50, CI = 1.167, 1.931), and men in Upper East (AOR = 2.625, CI = 1.608, 4.285) had higher odds of HIV testing uptake than their counter parts aged 15–19, those with no formal education unmarried and those in Western Region, respectively. However, men with no religion (AOR = 0.606, CI = 0.376, 0.975) and those who belong to the Mole-Dagbani ethnic group (AOR = 0.633, CI = 0.429, 0.934) had lower odds of HIV testing uptake compared to those who are Christians, and Akans, respectively. Men who have subscribed to health insurance (AOR = 1.896, 95% CI = 1.361, 2.643), those in the rich wealth quintile (AOR = 1.896, CI = 1.361, 2.643), those who read newspaper (AOR = 1.552, CI = 1.198, 2.012), listened to radio (AOR = 1.530, CI = 1.087, 2.153) at least once a week, and men who experienced discharge from their penis (AOR = 1.056, CI = 1.200, 1.515) had higher odds of HIV testing uptake.

**Conclusion:** Uptake of HIV testing among Ghanaian men is relatively low. There is the need for a concerted effort by various stakeholders to strengthen current efforts to target younger and unmarried men, men with low level of education, those who do not profess any religious affiliation and men belonging to Mole-Dagbani ethnic group.

## Introduction

Ghana has been seemingly successful in fighting against HIV, evident in the decline of the prevalence of the illness among the Ghanaian population. For instance, from 2003 to 2014, the prevalence of HIV among women aged 15–49 declined from 2.1 to 2.0%. A similar reduction of HIV prevalence was observed in the case of men of the same age group, with a decline of 2.8 to 1.1% in the same period of time (1). However, in order to meet the Joint United Nations Programme on HIV/AIDS (UNAIDS) 90-90-90 target by 2020, Ghana needs to work on the reduction further (2). This requires, among other things, an expansion of HIV testing services, so as to ensure that HIV-infected people are diagnosed and made aware of their status. In fact, some research attest to the effectiveness of HIV testing as a strategy for reducing HIV-induced deaths and illnesses (3, 4). Besides, the World Health Organization (WHO) and Center for Disease Control (CDC) also recognize the important roles played by HIV testing in fighting against the HIV pandemic (5, 6). For instance, when couples get to know their HIV status, they adopt strategies to avoid mother-to-child transmissions (7, 8). Also, when individuals get to know their HIV status, it helps them to get access to some medical and social support such as the antiretroviral therapy and emotional support needed to cope with HIV (9, 10).

Previous research reports low HIV testing among the general population in some low- and middle-income countries. In sub-Saharan Africa, the prevalence ranges from 69.9% in Malawi (11), 59% in South Africa (12), 25.1% in Tanzania (13), 23% in Uganda (10), and 22.7% in Ghana (14). Such studies have also reported low uptake of HIV testing services among males than among females, largely due to the emphasis placed on HIV testing among women prenatal care (15), who tend to use healthcare services more than men (16, 17). In the case of Ghana, an earlier study by Nyarko and Sparks (14) revealed a similar trend. This, therefore, highlights the need for further investigation of individual and contextual factors associated with uptake of HIV testing services among the general population in Ghana (18). Previous research on the Ghanaian situation has focused principally on women (13, 19–21), men sleeping with men (22), and young people (23). Generally, some studies have revealed an association between HIV testing among men and variables such as age (11, 12, 14, 24, 25), place of residence (11, 12, 14), marital status (11, 12, 14, 26), health insurance subscription (11), wealth status (27), educational level (11, 12, 14, 24, 26, 27), age at first sexual debut (11, 12), number of sex partners (25), condom use (24), religion (26), and media exposure (25).

In the present study, the factors associated with the uptake of HIV testing services among Ghanaian men were assessed. In Ghana, a patriarchal society, men usually lead the decision making process and control economic resources in households, which is likely to influence the control of economic resources that are significant for HIV prevention and care (11). It is, therefore, necessary to investigate the factors influencing the uptake of HIV testing in this population, as such factors will be needful in developing strategies to increase HIV testing among men in Ghana. For instance, findings from this study will reveal the specific category of men to be targeted with more efforts in order to improve HIV testing uptake. In fact, a similar study by Nyarko and Sparks (14) employed the 2003, 2008, and 2014 Ghana Demographic and health survey to explore the predictors of HIV testing among men in Ghana. The present study, however, differs from that study, since it focuses specifically on the 2014 version of the GDHS and also situates the study within an empirical model. This current study also considered only sexually active men. With this, it is hoped that the present study will extend the body of knowledge on HIV testing and counseling among men in Ghana and provide a better understanding of the phenomenon. The findings will augment existing efforts and strategies steering progress in the uptake of HIV testing among men in Ghana (14).

## Conceptual Framework

Evidence indicates that theory-based research is crucial in predicting human behaviors including utilization of sexual and reproductive health services such as HIV Testing (28). The study is underpinned by the Health Care Utilization Model originally propounded by Anderson and Newman (29). Over the years, the model has gone through successive modifications. For example, in 2000, Gelberg et al. modified the theory to include some challenges that impede healthcare access of vulnerable populations (30). There are three key elements in the model: predisposing, enabling, and need-for-care factors which either expedite or hinder the utilization of services by individuals (31). Predisposing factors include demographic characteristics, social structural variables, and an individual's basic beliefs, attitudes, and knowledge pertaining to health services (31). Enabling factors include resources available, whether individually or in a community (32). Need factors include the illnesses, conditions, and health statuses requiring health services. The model has been applied in various fields such as sociology, medicine, public health, and psychology. Specifically, it has been used to examine health care services utilization, such as HIV testing among young women in Trinidad and Tobago (33), American women in midlife (34), rural American cocaine users (35), primary care use among HIV-positive Haitian immigrants in Florida (36), racial/ethnic differences in HIV testing (32), and HIV testing service use among men in Haiti (37). Despite its wider application in different disciplines, some scholars have critiqued the model. Wilson et al. (38) were, for instance, of the view that the model does not pay attention to cultural dimensions and social interactions. Andersen (39), however, argued that need in itself is a social construct. In spite of the criticisms, the model is considered appropriate for this study because it is a multilevel theory and has been applied in various settings and disciplines.

## Materials and Methods

### Description of the Survey and Sampling

Data from the current, 2014 version of the Ghana Demographic and Health Survey (GDHS) were used for the study. The GDHS is conducted in five-year intervals nationwide, with specific focus on demographic and health issues, including HIV testing. Ghana Statistical Service and the Ghana Health Service are responsible for the conduct of the survey, with technical support from ICF International through MEASURE DHS. The sampling of participants for the survey is done at two stages. In the first stage, 427 Enumeration Areas(EAs) were chosen with probability proportional to the size of the EA with independent selection in each sampling stratum. The EA constitute the number of residential households inhabiting in the EA computed with the 2010 Population and Housing Census. A household listing operation was carried out in all the selected EAs, and the resulting lists of households served as a sampling frame for the selection of households in the second stage. To minimize the task of household listing for EAs with more than 200 households, each large EA was segmented. Only one segment was selected for the survey with probability proportional to the segment size. Household listing was conducted only in the selected segment. Therefore, a 2014 GDHS cluster is either an EA or a segment of an EA [1, p. 317–319]. At the second level of sampling, a fixed number of 30 households per cluster was selected with an equal probability systematic selection from the newly created household listing from January to March 2014. The survey interviewers visited and interviewed only the selected households. No replacements or changes of the selected households were allowed during data collection, in order to prevent bias. All women age 15–49 who were usual members of the selected households or who spent the night before the survey in the selected households were eligible for the female survey. In half of the selected households, all men age 15–59 who were usual members of the households or who spent the night before the survey in the households were eligible for the male survey. In all, the survey was conducted in 12,810 households, (6,480 urban and 6,330 in rural) [1, p. 317–319]. The total sample for the 2014 GDHS was expected to be 10,214 among women aged 15–49 (5,098 in urban and 5,116 in rural areas), and 4,175 completed interviews with men age 15–49 (2,061 in urban areas and 2,114 in rural areas). However, 4,388 men aged 15–59 were successfully interviewed. By extension, this implies a response rate of 95%. The present study featured 3,052 sexually active men because they had complete information on HIV testing, which the study is interested in. Detailed description of the methodology employed in the 2014 GDHS has been provided in the final report (1) which is also available online at https://dhsprogram.com/publications/publication-FR307-DHS-Final-Reports.cfm. Strengthening the Reporting of Observational Studies in Epidemiology' (STROBE) statement was followed in conducting this study and the writing of the manuscript.

## Study Variables

### Outcome Variable

In the present study, self-reported previous HIV testing was used as the outcome variable. The survey obtained information on this by asking this question: Have you ever tested for HIV? I considered HIV testing as a binary variable and categorized the responses into “Yes” or “No.” In the “Yes” category are those who reported ever testing for HIV at least once before the time of the survey. On the other hand, those who had never gone for HIV testing before fell under the “No” category.

### Independent Variables

**Figure 1:**
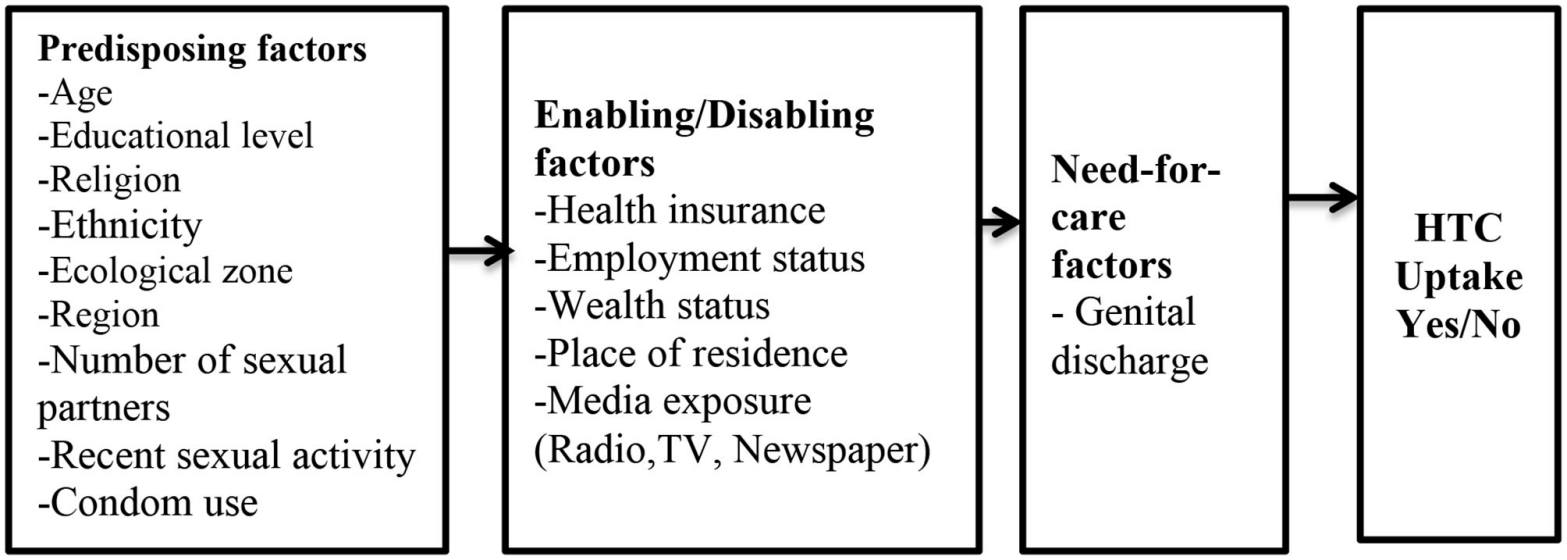
Conceptual Framework. Source: Anderson and Newman (29).

The independent variables were selected and grouped based on three reasons: first, conclusion from previous studies (11, 12, 14, 18, 19, 37, 40, 41) that have found them to have an association with HIV testing among men; second, the conceptual framework (Figure 1); and finally, their availability in the 2014 GDHS dataset.

### Predisposing Factors

Predisposing factors were categorized as follow: age (15–19, 20–29, 30–39, and 40 years or older), educational level (no education, primary, secondary, and higher), religious affiliation (Christianity, Islam, Traditionalist, and No religion), marital status (married and not married), ethnicity(Akan, Ga/Adangbe, Ewe, Mole–Dagbani, and Other), region (Western, Central, Greater Accra, Volta, Eastern, Ashanti, Brong-Ahafo, Northern, Upper East, and Upper West), zone (middle, northern and coastal), number of sex partners (1 and 2 or more), recent sexual activity (active and not active), and condom use during recent sexual activity with partner (yes and no).

### Enabling Factors

These include place of residence (rural and urban), employment status (working and not working), wealth index (poor, middle, and rich), health insurance subscription (yes and no), frequency of reading newspaper/magazine (not at all, less than once a week and at least once a week), frequency of watching television (not at all, less than once a week and at least once a week), and frequency of listening to radio (not at all, less than once a week and at least once a week).

### Need-For-Care

The need-for-care factor was captured as: genital discharge in the past 12 months (no and yes).

### Statistical Analyses

The analyses were done in three steps. At the initial stage, descriptive statistics (frequencies and percentages) was employed to describe the socio-demographic features of the men under investigation (see Table 1). Afterwards, bivariate analysis featuring Chi-square (χ2) was used to investigate the association between HIV testing and the independent variables. The variables that emerged statistically significant (*p* < 0.05) were transported to the next level, where multivariable logistic regression analysis was conducted to examine determinants of previous HIV testing. In the logistic regression modeling, three hierarchical logistic models were developed based on the categorization of the independent variables into predisposing factors, enabling factors, and need-for-care factors (see Table 2). The results were presented as adjusted odds ratios [AORs] with their respective 95% confidence intervals (CIs) indicating the level of precision. Prior to the regression analysis, multicollinearity among the variables were checked using variance inflation factor and there was no evidence of collinearity among the variables. STATA version 14.0 for Mac OS was used to conduct all the analysis and used the svy command to account for the complex sampling design. Weighting was also applied. Furthermore, the “svylogitgof” command was used to check for the fit of the logistic regression models, which showed no evidence of lack of fit of the models in significantly predicting HIV testing uptake among men in Ghana.

**Table 1:** HIV testing uptake by different background characteristics among sexually active men in Ghana.

**Variable**	***N*** **(3,052)**		**HIV testing status**		**χ2 (*****P*****-value)**	
	**Weighted**	**Weighted**	**Never tested**	**Ever tested**	
	***N***	**%**	**% (74.6%)**	**% (25.4%)**	
**Predisposing factors**					
**Age (years)**					23.07 (<0.001)
15–19	154	5.0	90.1	9.9	
20–29	818	26.8	75.9	24.1	
30–39	911	29.9	71.5	28.5	
40+	1,169	38.3	74.2	25.8	
**Educational level**					243.33 (<0.001)
No education	376	12.3	88.5	11.6	
Primary	390	12.8	84.4	15.6	
Secondary	1,889	61.9	74.0	26.0	
Higher	397	13.0	44.6	55.4	
**Religion**					57.67 (<0.001)
Christianity	2,207	72.3	70.8	29.2	
Islam	521	17.1	78.6	21.4	
Traditionalist	117	3.8	87.7	12.3	
No religion	208	6.8	88.3	11.7	
**Marital status**					14.5 (<0.001)
Not married	1,292	42.3	78.3	21.7	
Married	1,760	57.7	72.2	27.8	
**Ethnicity**					16.32 (0.003)
Akan	1,498	49.1	73.3	26.7	
Ga-Dangbme	289	9.5	70.2	29.8	
Ewe	431	14.1	70.0	30.0	
Mole-dagbani	417	13.7	77.4	22.6	
Others	417	13.7	79.3	20.7	
**Region**					36.61 (<0.001)
Western	365	12.0	74.9	25.1	
Central	304	10.0	74.3	25.7	
Greater accra	676	22.2	65.1	34.9	
Volta	242	7.9	74.3	25.7	
Eastern	294	9.6	73.2	26.8	
Ashanti	533	17.5	77.1	22.9	
Brong-ahafo	257	8.4	82.4	17.6	
Northern	218	7.1	80.8	19.2	
Upper east	99	3.2	70.1	29.9	
Upper west	63	2.1	73.5	26.5	
**Number of sex partners in the last 12 months**					1.02 (0.313)
1	2,436	79.8	75.0	25.0	
2 or more	616	20.2	73.0	27.0	
**Recent sex**					0.22 (0.640)
Active in last 4 weeks	2,034	66.6	74.3	25.7	
not active in last 4 weeks	1,018	33.4	75.1	24.9	
**Condom use**					10.56(0.001)
No	2,583	84.6	75.7	24.3	
Yes	469	15.4	68.7	31.3	
**Enabling factors**					
**Place of residence**					86.83(<0.001)
Urban	1,594	52.2	66.8	33.2	
Rural	1,458	47.8	81.5	18.5	
**Employment status**					2.64 (0.104)
Not working	117	3.8	80.8	19.2	
Working	2,935	96.2	74.3	25.7	
**Wealth index**					176.70 (<0.001)
Poor	974	31.9	83.8	16.2	
Middle	624	20.5	80.5	19.5	
Rich	1,454	47.6	61.3	38.7	
**Health insurance subscription**					111.07 (<0.001)
No	1,618	53.0	83.0	17.0	
Yes	1,434	47.0	66.4	33.6	
**Mass media exposure**					
**Frequency of reading newspaper /magazine**					193.94 (<0.001)
Not at all	1,994	65.3	81.5	18.5	
Less than once a week	527	17.3	64.3	35.7	
At least once a week	531	17.4	53.0	47.0	
**Frequency of watching television**					82.91 (<0.001)
Not at all	470	15.4	88.4	11.6	
less than once a week	563	18.4	75.0	25.0	
At least once a week	2,019	66.2	69.9	30.1	
**Frequency of listening to radio**					6.05 (0.041)
Not at all	127	4.2	82.7	17.3	
less than once a week	450	14.7	75.6	24.4	
At least once a week	2,475	81.1	73.9	26.1	
**Need-for-care factor**					
**Discharge from penis**					1.66 (0.042)
No	2,863	93.8	74.3	25.7	
Yes	189	6.2	78.4	21.6	

*GDHS 2014*.

**Table 2:** Multivariable logistic regression analyses of factors associated with HIV testing among sexually active men in Ghana.

**Variables**	**Model I AOR (95%CI)**	**Model II AOR (95%CI)**	**Model III AOR (95%CI)**
**Predisposing factors**			
**Age (Years)**			
15–19	Ref	Ref	Ref
20–29	2.229^**^ (1.245, 3.992)	2.323^**^ (1.274, 4.237)	2.327^**^ (1.276, 4.243)
30–39	2.774^***^ (1.517, 5.070)	2.706^**^ (1.453, 5.040)	2.715^**^ (1.458, 5.054)
40+	2.519^**^ (1.367, 4.645)	2.473^**^ (1.314, 4.652)	2.484^**^ (1.321, 4.673)
**Educational level**			
No education	Ref	Ref	Ref
Primary	1.562^*^ (1.054, 2.315)	1.443 (0.969, 2.149)	1.441 (0.967, 2.146)
Secondary	2.910^***^ (2.084, 4.064)	1.864^***^ (1.307, 2.660)	1.862^***^ (1.305, 2.656)
Higher	8.780^***^ (5.989, 12.87)	3.563^***^ (2.307, 5.502)	3.566^***^ (2.309, 5.509)
**Religion**			
Christianity	Ref	Ref	Ref
Islam	0.939 (0.711, 1.239)	0.87 (0.648, 1.168)	0.87 (0.648, 1.168)
Traditionalist	0.459^**^ (0.282, 0.749)	0.586^*^ (0.358, 0.960)	0.586^*^ (0.358, 0.959)
No religion	0.489^**^ (0.309, 0.775)	0.606^*^ (0.376, 0.976)	0.606^*^ (0.376, 0.975)
**Marital status**			
Not married	Ref	Ref	Ref
Married	1.634^***^ (1.286, 2.076)	1.497^**^ (1.166, 1.924)	1.501^**^ (1.167, 1.931)
**Ethnicity**			
Akan	Ref	Ref	Ref
Ga-Dangbme	0.963 (0.658, 1.409)	1.021 (0.686, 1.520)	1.02 (0.685, 1.518)
Ewe	1.166 (0.824, 1.649)	1.212 (0.841, 1.747)	1.212 (0.841, 1.746)
Mole-Dagbani	0.636^*^ (0.433, 0.934)	0.632^*^ (0.428, 0.933)	0.633^*^ (0.429, 0.934)
Others	0.796 (0.556, 1.140)	0.859 (0.599, 1.232)	0.859 (0.599, 1.231)
**Region of residence**			
Western	Ref	Ref	Ref
Central	0.976 (0.677, 1.408)	0.992 (0.677, 1.453)	0.992 (0.677, 1.453)
Greater accra	1.329 (0.929, 1.900)	1.07 (0.733, 1.560)	1.069 (0.733, 1.560)
Volta	0.946 (0.592, 1.514)	1.054 (0.646, 1.719)	1.052 (0.644, 1.717)
Eastern	1.088 (0.753, 1.570)	1.132 (0.772, 1.661)	1.131 (0.771, 1.659)
Ashanti	0.797 (0.550, 1.155)	0.706 (0.477, 1.046)	0.707 (0.478, 1.047)
Brong-ahafo	0.733 (0.504, 1.067)	0.801 (0.544, 1.180)	0.8 (0.543, 1.179)
Northern	1.359 (0.848, 2.178)	1.499 (0.928, 2.422)	1.492 (0.922, 2.414)
Upper east	2.519^***^ (1.581, 4.015)	2.623^***^ (1.606, 4.284)	2.625^***^ (1.608, 4.285)
Upper west	1.755^*^ (1.075, 2.865)	1.659 (0.997, 2.759)	1.653 (0.994, 2.751)
**Condom use**			
No	Ref	Ref	Ref
Yes	1.419^**^ (1.090, 1.846)	1.235 (0.940, 1.622)	1.237 (0.941, 1.625)
**Enabling factors**			
**Residence**			
Urban		Ref	Ref
Rural		0.986 (0.768, 1.266)	0.985 (0.767, 1.265)
**Wealth status**			
Poor		Ref	Ref
Middle		1.027 (0.758, 1.391)	1.027 (0.758, 1.391)
Rich		1.895^***^ (1.360, 2.642)	1.896^***^ (1.361, 2.643)
**Health insurance subscription**			
No		Ref	Ref
Yes		1.806^***^ (1.489, 2.192)	1.806^***^ (1.488, 2.191)
**Frequency of reading newspaper**			
Not at all		Ref	Ref
Less than once a week		1.268 (0.974, 1.650)	1.269 (0.975, 1.651)
At least once a week		1.553^***^ (1.198, 2.012)	1.552^***^ (1.198, 2.012)
**Frequency of watching television**			
Not at all		Ref	Ref
Less than once a week		1.674^**^ (1.169, 2.398)	1.674^**^ (1.169, 2.398)
At least once a week		1.531^*^ (1.088, 2.154)	1.530^*^ (1.087, 2.153)
**Frequency of listening to radio**			
Not at all		Ref	Ref
Less than once a week		1.03 (0.606, 1.752)	1.031 (0.606, 1.755)
At least once a week		0.934 (0.570, 1.531)	0.935 (0.570, 1.533)
**Need-for-care factors**			
**Discharge from penis**			
No			Ref
Yes			^*^1.056 (1.200, 1.515)
*N*	3,052	3,052	3,052
pseudo *R*^2^	0.099	0.135	0.136

*Computed from 2014 GDHS AOR, Adjusted odds Ratio*.

* *p < 0.05*,

** *p < 0.01*,

*** *p < 0.001; Ref, Reference*.

### Ethical Considerations

Ethical clearance was obtained from the Institutional Review Board of ICF International and Ethical Review Committee of Ghana Health Service. Demographic and Health Survey also anonymised all data before making them accessible to the public. Permission to use the data was obtained from MEASURE DHS, which is a USAID–funded project that assists and funds population and health surveys in countries worldwide.

## Results

### Characteristics of the Study Population and HIV Testing Uptake

Table 1 presents the weighted profile of men in the analysis sample by their HIV testing category. Of the 3,052 men interviewed, only about a quarter (25.6%) of them had tested for their HIV status. The age of the participants ranged from 15 to 59 years. Men aged 40 and above represented 38.3% of the sample. The majority (61.9%) of the respondents had secondary level of education. A greater percentage of the respondents were Christians (72.3%), married (57.7%), working (96.2%) and did not use condom (84.6%). Almost half (49.1%) of the sample were Akans. In terms of regional variations, men in the greater Accra region constituted 22.2% of the sample. Almost eighty percent of the men who participated in the study had one sexual partner in the past 12 months preceding the survey.

### Bivariate Analysis

The bivariate analysis showed that the predisposing factors associated with HIV testing are age, educational level, religion, marital status, ethnicity, region, and condom use. The enabling factors associated with HIV testing were place of residence, household wealth index, health insurance subscription, frequency of reading newspaper, frequency of watching television and frequency of listening to radio. Finally, the need-for-care factor associated with HIV testing was discharge from penis (see Table 1).

## Determinants of HIV Testing Uptake Among Sexually Active Men in Ghana

The determinants of HIV testing among men in Ghana are presented in Table 2. The predisposing factors such as age, educational level, religion, marital status, ethnicity, and region were found to be significantly associated with HIV testing. With age, it was found that men aged 30–39 had higher odds (AOR = 2.715, 95% CI = 1.458, 5.054) of HIV testing uptake, compared to men aged 15–19. HIV testing uptake increased with level of education. Specifically, men with higher level of education had 3.6 higher odds (AOR = 3.566, 95% CI = 2.309, 5.509) of HIV testing, compared to those with no formal education. With religion, it was shown that men with no religion (AOR = 0.606, 95% CI = 0.376, 0.975) had lower odds of HIV testing uptake, compared to those who are Christians. The enabling factors that were significantly associated with HIV testing were health insurance subscription and media exposure (reading newspaper and watching television). Specifically, men who had subscribed to health insurance had higher odds of HIV testing uptake (AOR = 1.896, 95% CI = 1.361, 2.643), compared to those who had not subscribed to national health insurance scheme. With media exposure, those who read newspaper (AOR = 1.552, 95% CI = 1.198, 2.012) and listened to radio (AOR = 1.530, 95% C I = 1.087, 2.153) at least once a week had higher odds of HIV testing uptake, compared to those who did not read newspaper and those who did not watch television. With the need-for-care factor, men who experienced discharge from their penis had higher odds (AOR = 1.056, 95% CI = 1.200, 1.515) for HIV testing uptake, compared to those who did not experience any discharge from their penis.

## Discussion

This study sought to assess the uptake of HIV testing services among sexually active men in Ghana. It was found from the study that only a quarter (25.4%) of sexually active men in Ghana had ever tested for their HIV status. The predisposing factors such as age, educational level, religion, marital status, ethnicity, and region demonstrated statistically significant association with HIV testing. The enabling factors that were significantly associated with HIV testing were health insurance subscription and media exposure (reading newspaper and watching television). With the need-for-care factors, men who experienced discharge from their penis had higher odds for HIV testing uptake, compared to those who did not experience any discharge from their penis.

The prevalence recorded in this study is similar to what has been found in previous studies in Ghana, other African settings and other low-and middle-income countries. For example, prevalence rate of 22.7, 23, 25.1, and 59% were found in Ghana (14), Uganda, (10), Tanzania (24), and South Africa (12), respectively. However, this current finding is lower than what was found in Zambia (61%) and Malawi (69%) by Hensen et al. (26) and Mandiwa and Namondwe (11), respectively. The low uptake of HIV testing reported in the present study could be attributed to the perception of low risk of transmission, mistrust of health professionals, and fear of HIV-related stigma, as well as the belief that once they look healthy and not engaging in any sexual activity, there is no need for HIV testing (14). The lower rate of men being tested for their HIV status indicates the need for prevention efforts to also target men since the predominant mode of HIV transmission is through heterosexual intercourse.

The study revealed a significant association between age and HIV testing among sexually active men in Ghana. With age, relative to adolescents, sexually active men who were aged 30–39 were more likely to test for HIV. Previous studies in Malawi (11), Haiti (37), South Africa (42) and Sierra Leone (43) reported similar findings. This finding could be due to the fact that adolescents might not have the courage to test for their HIV and AIDS status.

Educational attainment was found to be significantly associated with the odds of HIV testing. Specifically, the odds of HIV testing increased with the increase in educational attainment. This association is consistent with several previous studies (11, 14, 25, 43, 44). This finding is not surprising, as higher educational attainment has the likelihood of exposing individuals to health information, such as the importance of knowing one's HIV status. People with lower educational attainment, on the other hand, are less likely to get access to such information (11).

In line with some previous studies (14, 37, 45, 46), religious affiliation also showed significant association with HIV testing, with non-religious people showing less likelihood for HIV testing. This finding is suggestive of the role of religion in the fight against HIV/AIDS. USAID has attempted to use religious leaders as a channel to promote HIV testing. This finding may therefore be suggestive of the effectiveness of the efforts of UNAID in that regard (47). A study in Tanzania by Mbago (48), however, reported contrary findings. The differences in finding could be due to differences in socio-cultural settings and research methodologies adopted.

There was also a significant association between region of residence and HIV testing among Ghanaian men, with residents of Upper West Region being most likely to test for HIV. The findings concur with what has been reported in Ghana (14), Ethiopia (44), Malawi (11), and Mozambique (49). This finding suggests an influence of sociocultural differences that characterize different geographical regions on HIV testing (11). In the case of Ghana, northern Ghana has often been the focus of some non-governmental organizations aimed at improving the health of people. Corollary to the relationship between region of residence and HIV testing is the finding that ethnicity has a significant association with HIV testing among the studied population. Specifically, Mole-Dagbani men were less probable to go for HIV testing. A plausible explanation might be that there are strong cultural beliefs concerning certain procedures among this ethnic group, which might obstruct the uptake of these services. Nonetheless, there is a need for a further exploration of this relationship between HIV testing and ethnicity, perhaps through the use of qualitative methods, to reveal the reasons behind such an association.

Additionally, relative to unmarried men, married men were more probable to go for HIV testing. Previous studies in Kenya (50), Malawi (11), Haiti (37), and Mozambique (49) reported similar findings. This finding could be due to the fact that HIV testing is usually part of the preparations toward marriage (11). Evidently, in some African countries including Ghana, there are instances of mandatory premarital HIV testing (51), and this might explain the higher likelihood of HIV testing among married men reported in the present study. In addition, this difference in likelihood of HIV testing between married and non-married men seems to give credence to the assumption that marriage leads to changes in sexual behavior (37, 52).

In terms of household wealth, higher odds of HIV testing were reported among rich men than among poor men. This finding confirms previous studies in Ghana (14), Haiti (37), Uganda (53), Zimbabwe (46), and Burkina Faso (25). Relatedly, men who had subscribed to NHIS were more likely to test for HIV, compared to their counterparts who has not subscribed to NHIS. Previous studies in Haiti (37) and Malawi (11) similarly reported higher odds of HIV testing among men with health insurance. In line with the conceptual framework, household wealth and health insurance subscription are enabling factors to increase access to HIV testing services. In Ghana, clients with NHIS subscription are allowed free and regular access to HIV testing. Similarly, NHIS subscription increases men's use of health services which brings them into frequent contact with healthcare providers, which is likely to increase their likelihood of HIV testing (11).

Just like what was reported by some previous studies (14, 23, 25), mass media exposure also increased the likelihood of HIV testing among the studied men. In line with the conceptual framework (29), need-for-care factors such as discharge from penis increased the odds of service uptake. As rational beings, people with various symptoms of certain health conditions may put up behaviors to get rid of such conditions. It is, therefore, not surprising that men who experienced discharge from their penis had higher odds of testing for their HIV status.

## Strength and Limitations of the Study

Although this study has brought to bear some factors that determine HIV testing among sexually active men in Ghana that could have implications for policy, the limitations are not far-fetched. First, only variables that were collected in the GDHS were considered. As such, other important variables (such as sexual orientation) that may affect HIV testing but were not available could not be examined (11). Second, the cross-sectional nature of the GDHS makes it impossible to draw causal inferences but only associations (37). Third, there is the possibility of social desirability bias since the responses were self-reported (11, 37). Also, only data from the 2014 Ghana Demographic and Health Survey was used and as a result does not allow comparisons and evolution analysis. Despite these limitations, the study has its strength from the relatively large dataset and high response rate. The use of a nationally representative survey and the use of stratified two-stage sampling technique made it possible to obtain samples that are highly representative of the target population. The study also employed a behavioral model to guide the selection and interpretations of the findings. The large sample size and the national representativeness of the data, therefore, make the conclusions in this study more generalisable and valid.

## Conclusion

The prevalence of sexually active men who have ever tested for their HIV status was 25.4 percent, which is relatively low. The predisposing factors associated with HIV testing were age, educational level, religion, marital status, ethnicity, and region. The enabling factors for HIV status testing were wealth status, health insurance subscription, and media exposure (reading newspaper, watching television) while the need-for-care factor related to HIV testing is experiencing discharge from penis. There is the need for a concerted effort by various stakeholders to strengthen current efforts to target younger and unmarried men, men with low level of education, those who do not profess any religion and men belonging to Mole-Dagbani ethnic group. There is also the need to intensify HIV testing education in other regions of Ghana. These efforts can increase the number of men who seek HIV testing services and also encourage men to protect themselves and also could be made to encourage their partners and other members in their households to test and know their HIV and AIDS status. This might be a means for those who might be HIV-positive to benefit from antiretroviral treatment and, in turn, reduce the number of new infections, thereby helping to achieve the UNAIDS' 90-90-90 targets.

## Consent for Publication

This manuscript is an original work and has been done by the A-AS, and aware of its content and approved its submission. It is also important to mention that the manuscript has not been published elsewhere in part or in entirety and is not under consideration by another journal. The A-AS have given consent for this article to be submitted for publication in this Journal.

## Data Availability Statement

The datasets generated for this study will not be made publicly available the author does not have the power to share them.

## Author Contributions

A-AS conceived, analyzed the data, and drafted the manuscript.

## Conflict of Interest

The author declares that the research was conducted in the absence of any commercial or financial relationships that could be construed as a potential conflict of interest.
